# The role of Au–Cl adlayers in the Turkevich synthesis of gold nanoparticles

**DOI:** 10.1039/d6cc00457a

**Published:** 2026-02-23

**Authors:** Sarah May Sibug-Torres, Gregory Q. Wallace, Tabitha Jones, Duncan Graham, Jeremy J. Baumberg

**Affiliations:** a NanoPhotonics Centre, Cavendish Laboratory, Department of Physics, JJ Thompson Avenue, University of Cambridge Cambridge CB3 0US UK jjb12@cam.ac.uk; b Centre for Nanometrology, Department of Pure and Applied Chemistry, Technology and Innovation Centre, University of Strathclyde Glasgow G1 1RD UK

## Abstract

Real-time surface-enhanced Raman spectroscopy reveals that Au–Cl adlayers modulate the surface chemistry of gold nanoparticles during Turkevich synthesis. The formation, evolution, and collapse of this adlayer account for the characteristic optical, electrochemical, and size changes observed during growth and termination, offering a unified chemical picture of gold nanoparticle synthesis.

## Abstract 2

Real-time surface-enhanced Raman spectroscopy reveals an intermediate Au–Cl surface adlayer that controls growth and termination during gold nanoparticle synthesis.

## Graphical abstract

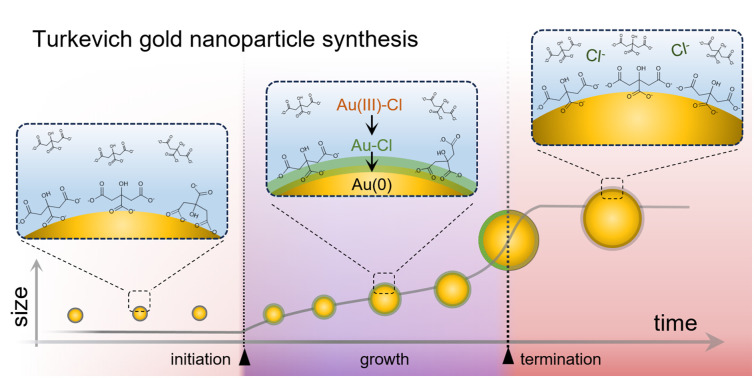

The Turkevich method, introduced in the 1950s, remains one of the most widely used routes for synthesizing gold nanoparticles (AuNPs).^1^ By reducing HAuCl_4_ with citrate under aqueous conditions, it provides a simple and reproducible synthesis that has driven decades of advances in plasmonics, catalysis, sensing, and biomedicine.^2,3^ Despite its ubiquity, many of its mechanistic details have remained unclear for decades as most studies rely on *ex situ* characterization of intermediates, which often introduce artefacts such as aggregate structures.^4–7^ A major shift came from the work of Polte *et al.*, who employed *in situ* SAXS/XANES to track particle size evolution during synthesis.^2,8^ Their data revealed a seed-mediated process in which Au nuclei form, coalesce into stable seeds, and subsequently grow through the surface reduction of Au(iii) precursors. These insights were only accessible under operando conditions where artifacts from drying and electron beam-induced restructuring and reduction are avoided.^4–7^

Even with this improved growth model, notable optical and electrochemical features of the Turkevich synthesis remain unexplained. During growth, the reaction mixture evolves from yellow to blue and purple before suddenly turning red, suggesting that the localized surface plasmon resonance (LSPR) exhibits a progressive blue shift even as the AuNPs increase in size^1,4,7^ (Fig. S1), an inversion of the typical size-LSPR relationship.^9^ To rationalize this anomaly, it was proposed that the growing AuNPs acquire a transient shell that modifies their optical response,^2,8^ potentially arising from AuCl_4_^−^ retained within the electrical double layer.^2,3^ Support of this hypothesis came from regrowth experiments by Polte^2,10^ and Panariello,^11^ where injection of HAuCl_4_ into citrate-stabilized seeds caused an initial red shift, followed by a gradual then sudden blue shift as growth progressed (Fig. 1a). Optical simulations further suggested that a low-conductivity high-damping shell could reproduce these spectral trends.^11^ These studies implied that the evolving LSPR is shaped not only by the particle size but also by its surface chemistry. However, this precise surface chemistry has remained unknown and inaccessible using conventional techniques.

**Fig. 1:**
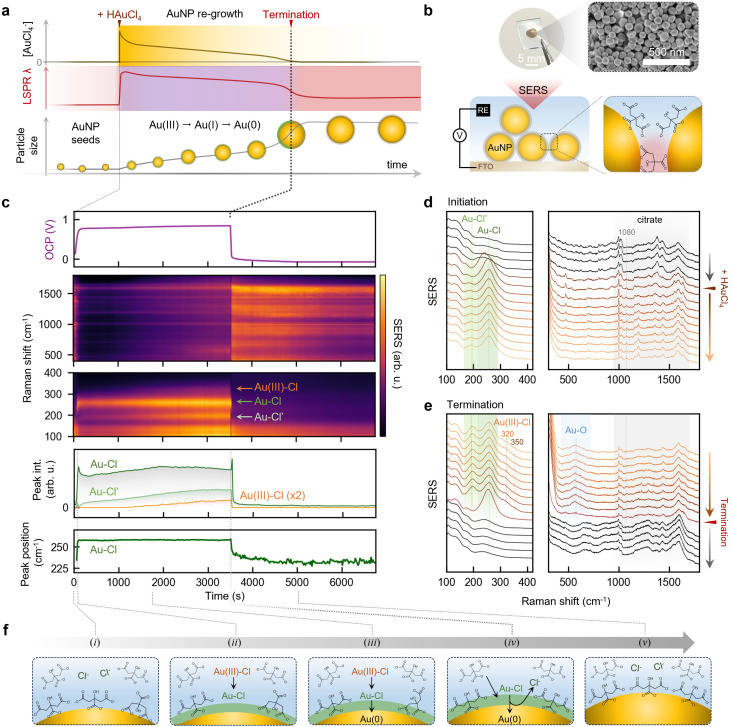
Real-time SERS and OCP tracking during simulated AuNP regrowth on MLagg-citrate. (a) Compilation of key observables reported in the literature during AuNP regrowth, including bulk AuCl_4_^−^ concentration, LSPR of AuNPs, and nanoparticle size evolution. To initiate regrowth, AuNP seeds in citrate solution are treated with HAuCl_4_, resulting in a red-shift or decrease in wavelength (*λ*) of the AuNP LSPR. Growth proceeds with the gradual consumption of bulk AuCl_4_^−^, blue shift of LSPR, and increase in AuNP size. Termination is characterised by the depletion of AuCl_4_^−^, sharp blue shift in LSPR, and a rapid increase in AuNP size. (b) MLagg-citrate platform. Photograph (left) and SEM (right) of the MLagg-citrate deposited on FTO. Schematic below shows aggregated AuNPs forming sub-nm SERS-active nanogaps on FTO, coated with citrate ligands and integrated into a spectro-electrochemical cell for simultaneous SERS and OCP measurements. (c) Real-time open-circuit potential (OCP, purple), SERS spectra, and extracted peak areas of Au–Cl features (indicated in the SERS spectra). Lower panel shows the evolution of the Au–Cl peak position with time. (d) and (e) SERS spectra at (d) the onset of growth, and (e) near growth termination highlighting Au–Cl and citrate modes. Key spectral features are labelled. (f) Proposed interfacial chemistry inferred from MLagg-citrate SERS data, showing (i) initial citrate coating, (ii) formation of a Au–Cl adlayer upon HAuCl_4_ addition, (iii) reduction of Au(iii)–Cl to Au–Cl and further to Au(0) during growth, and the (iv) final reduction of Au–Cl to Au(0) and (v) re-establishment of citrate passivation at the termination of growth.

A second equally longstanding mystery arises at growth termination. Classical studies consistently report a rapid increase in particle size, a sharp LSPR blue shift, and a collapse of the open-circuit potential (OCP) from +0.7 V to +0.1 V (*vs*. Ag/AgCl) at growth termination.^3,7,8^ The concurrence of these optical, morphological, and electrochemical signatures suggests a collective and rapid restructuring at the nanoparticle surface, yet the underlying trigger for this transformation has likewise remained unidentified.

In this work, we address these gaps by constructing a surface-confined analogue of colloidal AuNP seeds, enabling direct spectroscopic observation of the interfacial chemistry during simulated AuNP growth and termination. Using citrate-coated close-packed films of AuNPs (a multi-layer aggregate termed ‘MLagg-citrate’) as immobilized seed analogues, we introduce HAuCl_4_ under Turkevich-like regrowth conditions and track the interfacial chemistry using real-time surface-enhanced Raman spectroscopy (SERS) and OCP measurements. This approach expands upon the electrochemical model of Rodríguez-González,^7^ who correlated LSPR evolution, bulk HAuCl_4_ depletion, and the OCP of a Au electrode immersed in a AuNP synthesis mixture. Here, MLaggs offer the additional advantage of direct spectroscopic access to the nanoparticle surface throughout growth and termination. By combining real-time SERS with electrochemical tracking, we uncover a Au–Cl adlayer as the active interfacial species during growth, and show how its destabilization triggers the abrupt transition that terminates particle growth. By linking real-time tracking of interfacial chemistry to decades of observations in AuNP synthesis, this work provides a unified mechanistic framework for both the anomalous LSPR behaviour during growth and the sudden growth acceleration at termination in the Turkevich method.

To resolve these interfacial transformations in real time, we first prepare a well-defined MLagg-citrate platform. Aggregated 80 nm AuNPs are deposited as a near-monolayer film on fluorine-doped tin oxide (FTO) glass (Fig. S2a and b), forming sub-nanometre gaps that function as SERS hotspots optimised for enhancement under 785 nm laser excitation.^12,13^ This MLagg-film is subjected to electrochemical cleaning to remove initial surface species and to form a Au oxide layer, and finally re-scaffolded^14^ to generate a reproducible citrate-terminated surface (Fig. S2c). The MLagg-citrate is then assembled into a spectro-electrochemical cell to enable simultaneous OCP and SERS tracking (Fig. S3). At this stage, SERS confirms that citrate adopts multiple binding modes on the Au surface, including gap-spanning geometries.^15^

To mimic AuNP seed re-growth conditions^10,11^ at room temperature, the cell is filled with 1 mM citrate (adjusted to pH 5–6 with HCl) and allowed to equilibrate at an OCP of +0.3 V *vs.* Ag/AgCl. Growth is then initiated by injecting HAuCl_4_ to a final concentration of 10 µM, and SERS spectra are collected every 20 s with minimal laser exposure to avoid photothermal effects. A low HAuCl_4_ concentration is used to minimise nanogap sintering from excessive Au deposition.

Upon HAuCl_4_ injection into the citrate solution, the MLagg-citrate exhibits an immediate and pronounced change in both SERS and OCP. A strong band appears at 260 cm^−1^, consistent with the formation of surface-bound Au–Cl (Fig. 1c and d), while the OCP rises to +0.7 V. A weaker band also emerges at ∼175 cm^−1^ and gradually shifts towards 200 cm^−1^. Such low-wavenumber features can arise either from Cl–Au(iii)–Cl bending modes from Au(iii)–Cl species or from Cl^−^ bound to Au adatoms.^16^ However, the dominant SERS feature is the 260 cm^−1^ mode, and no Au(iii)–Cl stretching band at ∼350 cm^−1^ is detected, indicating that Au(iii) species do not persist and that the interface becomes rapidly dominated by Au–Cl upon mixing HAuCl_4_ with citrate (Fig. 1f(ii)).

The nature of this metastable surface coating is central to understanding the anomalous growth behaviour of AuNPs. Early models treated the growth-phase interface as positively charged Au with electrostatically bound Cl^−^,^4,7^ whereas atomic force measurements instead identified an uncharged steric surface layer of unknown chemical identity during growth.^17^ Subsequent studies have postulated the involvement of Au–Cl complexes as transient organisers during nucleation.^18^ However, these species have not yet been directly observed in real-time, nor linked to the optical, electrochemical, or growth-termination behaviour of AuNPs.

Here, our data directly identify this metastable surface coating as a covalent Au–Cl adlayer. In our recent detailed characterisation,^16^ we showed that this adlayer forms when surface Au atoms are oxidised to Au(i) and bind Cl^−^ through a covalent interaction. The resulting Au–Cl bonds impose a strong outward-facing surface dipole that withdraws electron density from the Au surface and elevates the work function, a behaviour directly reflected in the +0.7 V OCP established during growth.^16^ This Au–Cl adlayer matches the low-conductivity shell proposed by Panariello to explain the LSPR evolution of growing AuNPs.^11^ Consistent with this assignment, dark-field scattering from MLagg-citrate shows that immediately after HAuCl_4_ addition, the coupled plasmon mode decreases in intensity and red shifts (Fig. S4), mirroring the LSPR response reported for colloidal seeds.^2,3,11^ These observations identify the Au–Cl adlayer as the likely surface-bound electron-deficient layer responsible for the optical signatures of AuNPs during growth.

The kinetic evolution of the interface further corroborates the assignment of Au–Cl as the dominant surface species during growth. The sharp rise in the Au–Cl band, together with the absence of Au(iii)–Cl features, reflects the rapid initial consumption of AuCl_4_^−^ reported by Rodríguez-González.^7^ Both citrate-mediated reduction of AuCl_4_^−^ to Au(i) (eqn (1)) and comproportionation of surface Au(0) with AuCl_4_^−^ likely contribute to the instantaneous formation of the Au–Cl adlayer, consistent with the fast Au(iii) → Au(i) conversion established in kinetic studies.^19^ This latter pathway is supported by control experiments in which MLagg-citrate is exposed to HAuCl_4_ in the absence of bulk citrate, yielding coexisting Au(iii)–Cl features and a 260 cm^−1^ Au–Cl band (Fig. S5). The simultaneous presence of these signals indicates that in the absence of bulk citrate, formation of Au(i) from AuCl_4_^−^ can proceed through the oxidation of surface Au(0). In contrast, the subsequent Au(i) → Au(0) reduction is rate determining (eqn (2)).^19^ These signatures confirm that when HAuCl_4_ is added to citrate, the resulting interface is dominated by a surface-bound Au–Cl adlayer, rather than Au(iii) species or soluble Au(i)Cl_2_^−^ complexes (Table S1). Au–Cl thus emerges as the principal Au(i) surface intermediate during growth, mediating Au(0) atom deposition from Au(iii) precursors.

1 Citrate + AuCl_4_^−^ → 1,2-dicarboxyacetone + Au–Cl + H^+^ + 3Cl^−^ + CO_2_

2 3Au–Cl + Cl^−^ → 2Au(0) + AuCl_4_^−^

As growth proceeds, the Au–Cl band declines sharply but equilibrates, indicating the reduction of surface Au–Cl to Au(0) is balanced by its continued replenishment from AuCl_4_^−^. The decrease in the Au–Cl band is accompanied by a rise in the 1080 cm^−1^ citrate-adatom binding mode^15^ and an increase in SERS background, consistent with deposition of new Au(0) atoms and associated nanogap surface roughening^20^ (Fig. S5, Table S2 and SI Note 1). At this stage, the OCP remains near +0.7 V, a state that Rodríguez-González previously associated with the continued presence of AuCl_4_^−^ in the bulk solution.^7^ We thus infer that appreciable AuCl_4_^−^ persists during mid-growth, enabling ongoing regeneration of the Au–Cl adlayer through citrate-mediated reduction of AuCl_4_^−^ (eqn (1)). Mid-growth is thus characterised by a dynamic Au(iii)–Cl → Au(i)–Cl → Au(0) cycle in which surface Au–Cl is reduced to deposit Au(0) while new Au–Cl forms from Au(iii) species originating from bulk HAuCl_4_ (eqn (1)) and those generated from the disproportionation of Au–Cl (eqn (2)).

As the reaction approaches full depletion of HAuCl_4_, a distinct late-growth stage emerges (Fig. 1c and e).^4,7,21^ Au–Cl begins to decline once more, while weak Au(iii)–Cl (320, 350 cm^−1^) and Au–O (∼550 cm^−1^) features emerge. These spectral changes, together with a gradual rise in OCP from +0.7 V to +0.8 V, suggest that the diminishing bulk HAuCl_4_ concentration slows the regeneration of the Au–Cl adlayer. Under these conditions, the rate of Au–Cl disproportionation to form Au(0) and higher-valence Au species (eqn (2)) outpaces Au–Cl generation from AuCl_4_^−^ (eqn (1)).

Once the supply of HAuCl_4_ becomes insufficient to sustain adlayer regeneration, the system enters a critical transition. Immediately beforehand, the remaining Au(iii) species are rapidly reduced by excess citrate, producing a brief surge in the Au–Cl band that reflects a final regeneration of the adlayer from the last accessible Au(iii) species. Without continued precursor supply and under reducing conditions, this newly formed Au–Cl layer is intrinsically unstable. The system then undergoes a sharp and spontaneous collapse: within 30 s, the Au–Cl band drops sharply and shifts from 260 to 240 cm^−1^ (indicating decreasing adlayer density^16^), the OCP falls from +0.8 V to +0.1 V, and the SERS background surges. This collapse encompasses a rapid self-amplifying breakdown of the Au–Cl dipole layer. At the onset of this breakdown, the surface becomes increasingly electron-rich, which further destabilises the remaining Au–Cl and accelerates its reduction. This internal feedback drives the reduction cascade: a lower work function enhances the driving force for reducing residual Au–Cl, which in turn lowers the work function further, hastening the loss of the adlayer. Citrate then binds to the now Au(0) surface as the Cl^−^ is removed, generating broad disordered citrate features that later sharpen as the surface reorganises into a stable citrate-passivated state (Fig. 1f(iv and v)). The marked rise in SERS background and adatom (‘picocavity’) signatures^20^ highlights intense atomic-scale restructuring during this transition.

Characterisation of surface chemistry using the MLagg-citrate during AuNP growth and termination resolves several longstanding aspects of the Turkevich method. Although the MLagg-citrate architecture senses chemistry under nanoscale confinement and operates under room-temperature static conditions (SI Note 2), the redox sequence and interfacial transformations observed here closely parallel colloidal growth behaviour in solution. Earlier studies reported a rapid reduction cascade, a final burst of Au(0) deposition, Cl^−^ expulsion, and an abrupt LSPR blue shift at termination,^3,7,8^ yet the chemical origin of these transitions remained unresolved. By directly observing the formation, evolution, and collapse of the Au–Cl adlayer (Fig. 1f), we show that this covalent electron-withdrawing shell shapes the surface redox potential, growth kinetics, and ligand dynamics throughout synthesis. During growth, a dynamic balance between Au–Cl regeneration and its reduction to Au(0) maintains a high-work-function state. As HAuCl_4_ becomes depleted, this balance collapses: the adlayer destabilises, the surface potential drops, and a self-reinforcing reduction cascade is triggered. The loss of the electron-deficient Au–Cl shell removes the barrier to rapid Au(0) deposition, explaining the sudden size increase observed by SAXS,^8^ while the accompanying change in surface electron density accounts for the sharp solution colour change from purple to red. Thus, termination arises from a redox-driven destabilisation and collapse of the Au–Cl adlayer, providing a unified chemical basis for the kinetic, structural, and optical signatures that define the endpoint of AuNP growth.

These insights position the Au–Cl adlayer as a redox-active growth mediator whose stability and collapse determine the trajectory and endpoint of AuNP formation. Given that this behaviour arises from a halide-stabilised Au(i) surface intermediate, an important question is whether other halides produce analogous interfacial states. Substitution of HAuCl_4_ with HAuBr_4_ in AuNP synthesis shows markedly distinct growth behaviour (SI Note 3), consistent with the formation of covalent Au-X surface species whose halide-dependent stability^22^ reshapes the kinetics of the shared Au(iii) → Au(i)–X → Au(0) pathway, thereby influencing growth rates and facet evolution.^23^ Thus, more broadly, modulating the stability of this interfacial layer may offer new opportunities for controlling nanoparticle size, growth rate, and optical response across diverse AuNP synthesis protocols.

S. M. S.-T. and J. J. B. conceived and designed the experiments. S. M. S.-T. carried out the optical and electrochemical experiments. G. Q. W. carried out AuNP synthesis and characterisation. All authors contributed to the paper writing and/or editing.

## Conflicts of interest

The authors declare the following competing interests: Cambridge Enterprise, Ltd has filed a patent application (WO2024199899A1, pending) on which J.J.B. and S.M.S.-T. are listed as inventors. All other authors have no conflicts to declare.

## Supplementary Material

CC-062-D6CC00457A-s001

## Data Availability

The underlying data that support the findings of this study are available from the main text, its supplementary information (SI) or the Cambridge Open Data archive at https://doi.org/10.17863/CAM.127283, and from the corresponding author upon request. The supplementary information contains detailed methods, additional spectroscopic data, tabulated summaries of spectral features, and extended mechanistic discussion supporting the proposed AuNP regrowth model. See DOI: https://doi.org/10.1039/d6cc00457a.
