# Supplementary material for: The role of Au–Cl adlayers in the Turkevich synthesis of gold nanoparticles
Source: Chem Commun (Camb). 2026 Feb 23;62(21):5890–4. doi: 10.1039/d6cc00457a (PMC12947627; doi:10.1039/d6cc00457a)
Supplement: CC-062-D6CC00457A-s001 [file CC-062-D6CC00457A-s001.pdf]

Supplementary Information (SI) for ChemComm.
This journal is © The Royal Society of Chemistry 2026

Electronic Supplementary Information

The role of Au-Cl adlayers in the Turkevich synthesis of gold nanoparticles

Sarah May Sibug-Torres[1], Gregory Q Wallace[2], Tabitha Jones[1],
Duncan Graham[2], Jeremy J Baumberg[1*]

[1] NanoPhotonics Centre, Cavendish Laboratory, Department of Physics, JJ Thompson Avenue,
University of Cambridge, Cambridge, CB3 0US, UK
[2] Centre for Nanometrology, Department of Pure and Applied Chemistry, Technology and Innovation Centre,
University of Strathclyde, Glasgow G1 1RD, UK
* Corresponding author: jjb12@cam.ac.uk

Methods 2
SI Note 1: Evolution of citrate binding modes during AuNP growth 9
SI Note 2: Limitations of MLagg-citrate model 13
SI Note 3: Preliminary AuNP synthesis with $AuBr_4^-$ 14
References 16

Supplementary Figures

Fig. S1. Schematic summary of seed-mediated AuNP growth model in the Turkevich synthesis. 4
Fig. S2. Preparation, oxidative cleaning, and re-scaffolding of MLagg-citrate substrates. 5
Fig. S3. Spectro-electrochemical cells for static measurements. 6
Fig. S4. Dark field scattering spectra of MLagg-citrate before and after adding $HAuCl_4$. 7
Fig. S5. MLagg-citrate in $HAuCl_4$ with and without citrate. 8
Fig. S6. Interfacial transformations during simulated AuNP regrowth on MLagg-citrate. 10
Fig. S7. Comparison of AuNPs prepared by $HAuCl_4$ and $HAuBr_4$ 14
Fig. S8. Scanning electron microscope images of AuNPs prepared using $HAuBr_4$ 14
Fig. S9. Comparison of AuNP seeds prepared with $HAuCl_4$ and $HAuBr_4$ 15

Supplementary Tables

Table S1 | Raman modes for surface-bound Au species and citrate 11
Table S2 | Summary of citrate-based AuNP regrowth stages. 12

Methods

Materials

All chemical reagents were used as received. Citrate-stabilised AuNPs (80 nm, optical density 1.0 at 555 nm, ≤8% coefficient of variation, suspended in H_2O with residual chemical from synthesis) were from BBI Solutions. Trisodium citrate (≥99%) and $HAuCl_4 \bullet 3H_2O$ (≥99.9% metals basis) were from Sigma Aldrich. $HAuBr_4 \bullet xH_2O$ (99.99% metals basis) was purchased from Thermo Scientific. Analytical grade chloroform, HCl (37%), and H_3PO_4 (86%), were from Fisher Scientific. NaCl (≥99%), K_2HPO_4 (≥98%), and KH_2PO_4 (≥98%) were from Alfa Aesar. NaOH (≥98%) was from Acros Organics. Polydimethylsiloxane (PDMS) slabs were prepared using a SYLGARD 184 kit from DOWSIL (Dow Silicones). Fluorine-doped tin oxide (FTO)-coated glass slides (TEC 10, 45 nm surface roughness) were purchased from Ossila Ltd and were cleaned and cut to 10 x 7.5 mm^2 slides prior to use. All aqueous solutions were prepared using deionized (DI) water (≥0.5 MΩ·cm) from Thermo Fisher Scientific.

Multilayer aggregate preparation

MLagg substrates were prepared[1–3] by mixing 500 µL citrate-stabilised, aqueous-based 80 nm AuNPs[†] with equal volume chloroform, and initiating aggregation with the addition 50 µL of 1 M NaCl. Aggregation was facilitated with 1 min of vigorous shaking, after which the aggregates settled at the liquid-liquid interface. The AuNP aggregates were then washed with DI water to remove residual ligands and salts by repeatedly (at least 3x) concentrating and diluting the aqueous phase, followed by a final concentration step in which the volume was reduced to ~5 µL. The AuNP aggregate was then transferred via pipette to a cleaned FTO-coated glass slide and allowed to air dry (**Fig. S2a-b**). The deposited MLagg was further rinsed with DI water to remove residual ligands and salts, and subsequently dried with compressed N_2.

Cleaning and re-scaffolding

Native AuNP ligands and surfactants were removed from the freshly prepared MLagg surface with electrochemical oxidation by applying of +1.5 V for at least 30 s in 1 M potassium phosphate buffer (pH 7.0). After oxidative cleaning, citrate was introduced by applying a linear sweep potential from +0.7 V to 0 V at 25 $mV \cdot s^{-1}$ in 1 M citrate and 0.5 M HCl (**Fig. S2c**).

Raman and dark-field measurements

Raman spectra were taken on a Renishaw inVia Raman microscope with 785 nm excitation laser using a ×20 NA 0.40 objective. Time-series spectra were collected with a laser power of 2.2 mW and an integration time of 1 s, with 20 s between each spectrum. Laser shutter was closed between each measurement to minimise laser exposure onto the sample between measurements. Additional Raman measurements were collected using a custom set-up[3] with an Andor Newton 970 EMCCD camera coupled to a Shamrock 168 spectrometer and a Matchbox 785 nm diode laser. Excitation and collection were collected through an Olympus LUMPlanFl/IR ×40 W NA 0.80 water-immersion objective at 1 s integration times with 1 mW laser power.

DF scattering measurements were collected using a modified Olympus BX51 with an Ocean Optics QE-Pro spectrometer. Excitation and collection were performed through an Olympus MPLanFL N ×20 BD NA 0.45 objective with 500 ms integration time. A white light scattering target (Labsphere) was used as a reference to normalize white light scattering.

EC-SERS and EC-DF set-up

Spectro-electrochemical cells were designed and fabricated to measure time-resolved SERS and DF scattering spectra with simultaneous electrochemical measurements. An EC-SERS cell was fabricated from PDMS to accommodate a three-electrode electrochemical system: a Pt wire (Sigma-Aldrich) counter electrode, a leakless Ag/AgCl/KCl (LF-1-45 from Innovative Instruments Ltd.) reference electrode, and a MLagg SERS substrate on FTO-coated glass as the working electrode. The electrolyte compartment was defined using a 6 mm diameter biopsy punch. Using custom 3D-printed stage holders, the EC-SERS cell was sealed and mounted onto the stage of an inverted Raman set-up, with SERS probed from below the cell. To measure time-resolved EC-DF and EC-SERS spectra on upright microscope set-ups, another spectro-electrochemical cell was machined from Teflon to accommodate the same electrochemical system. The EC-DF cell was mounted below the objective, with DF

[†] 80 nm AuNPs were selected to optimise SERS enhancement under fixed 785 nm excitation; smaller particles (≤20 nm) scatter weakly and require extended chain growth to achieve comparable near-infrared coupling, and were found to give lower signal and less reproducible film formation under our deposition conditions.[4,5]

scattering measured through a 0.13-0.16 mm coverslip and a thin (0.5 mm) layer of electrolyte. Electrochemical measurements were conducted using a portable potentiostat (CompactStat) from Ivium Technologies in static conditions. For OCP measurements, only two electrodes (WE and RE) were integrated into the cell (**Fig. S3**). All potentials were referenced to the Ag/AgCl reference electrode.

Data analysis

SERS spectra were background-corrected, where applicable, to remove the broad glass signal centred at 1400 cm^{-1} from back-side optical measurements. SERS peak areas were determined by first defining a spectral region, fitting a polynomial background, and fitting Gaussian curves to the peaks of interest.

$AuCl_4^-$ / $AuBr_4^-$ AuNP synthesis methods

Three methods for synthesizing AuNPs were considered. For each, a comparison between using $HAuCl_4$ and $HAuBr_4$ as the gold precursors was made to ascertain the effectiveness of substituting the precursor. For both methods, the gold content was maintained with the assumption that for $HAuBr_4$•xH_2O, x = 5.

In the first method, the gold precursor (20.8 mg for $HAuCl_4$, or 32.1 mg for $HAuBr_4$) was added to a cleaned 250 mL round bottom flask, and dissolved in 150 mL of doubly distilled deionized water (gold concentration of 0.352 mM). The solution was brought to a boil under reflux conditions using an oil bath. After 10 minutes of boiling, 12.5 mg of citrate dissolved in 1 mL of doubly distilled deionized water (42.8 mM) was rapidly added. Boiling was maintained for 15 minutes, before the solution was allowed to cool to room temperature. The synthesis was repeated for the other gold precursor. In the second method, the same quantities were used, but the addition sequence was reversed. Instead, the citrate was dissolved in 150 mL of doubly distilled deionized water, and the gold precursors dissolved in 1 mL of doubly distilled deionized water.

The third method took inspiration from the seed AuNP synthesis of Bastus *et al*.[6] Here, 97.1 mg of trisodium citrate dihydrate was added to a cleaned 250 mL round bottom flask, and dissolved in 150 mL of doubly distilled deionized water (citrate concentration of 2.2 mM). The solution was brought to a boil under reflux conditions using an oil bath. After 10 minutes of boiling, 1 mL of a 25 mM gold precursor solution (9.8 mg for $HAuCl_4$, or 15.2 mg for $HAuBr_4$ dissolved in 1 mL of doubly distilled deionized water) was rapidly added. Boiling was maintained for 15 minutes, before the solution was allowed to cool to room temperature. The synthesis was repeated for the other gold precursor.

Characterization of Synthesized AuNPs

Synthesized AuNPs were characterized by extinction spectroscopy with a Cary 60 UV–vis-NIR spectrometer, scanning from 300 to 1100 nm at a scan rate of 4800 nm min^{-1}. Scanning electron microscopy (SEM) imaging was carried out using an FEI Quanta 250 FEG scanning electron microscope from Oxford Instruments, with a voltage of 5 kV and a spot size of 2.0. Samples were prepared by spotting 1 µL of sample onto a piece of silicon wafer and allowing it to dry.

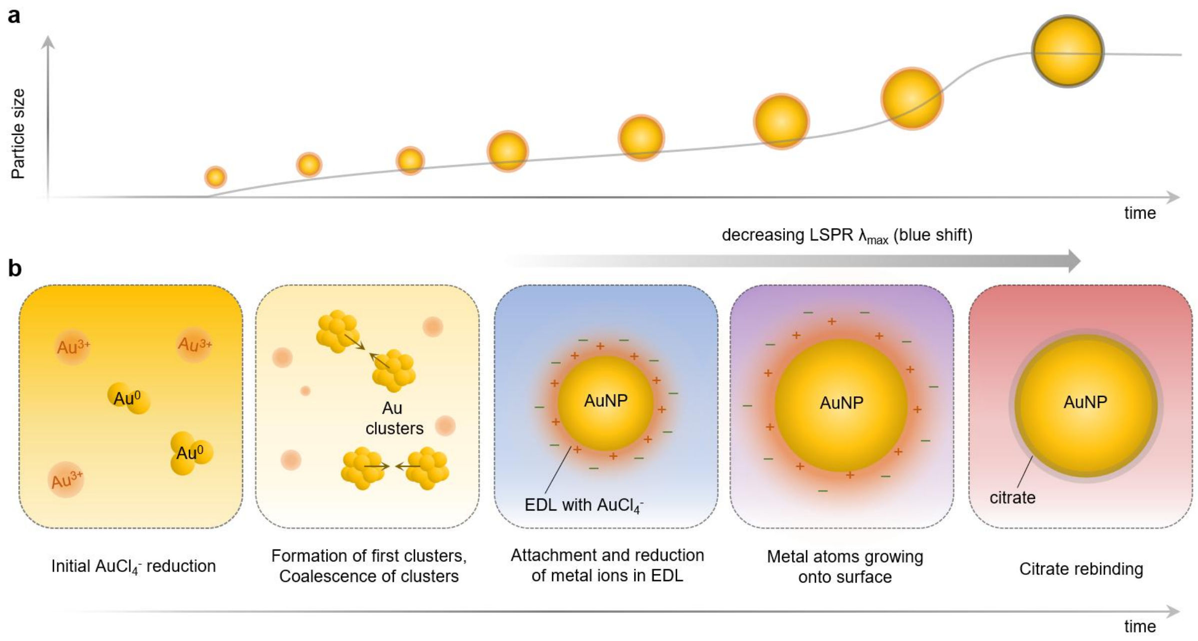

Fig. S1. Schematic summary of seed-mediated AuNP growth model in the Turkevich synthesis. (**a**) Growth model adapted from Polte *et al.*, showing initial Au nuclei formation, their coalescence into stable seeds, and subsequent particle growth via surface reduction of Au precursor species[7]. (**b**) Panels illustrate the growth of AuNPs throughout the distinct solution colour changes, from yellow ($HAuCl_4$ precursor and Au clusters) to blue and purple (early-stage AuNPs with an electrical double layer hypothesized to contain $AuCl_4^-$), and finally to red (mature AuNPs stabilized by surface-bound citrate).

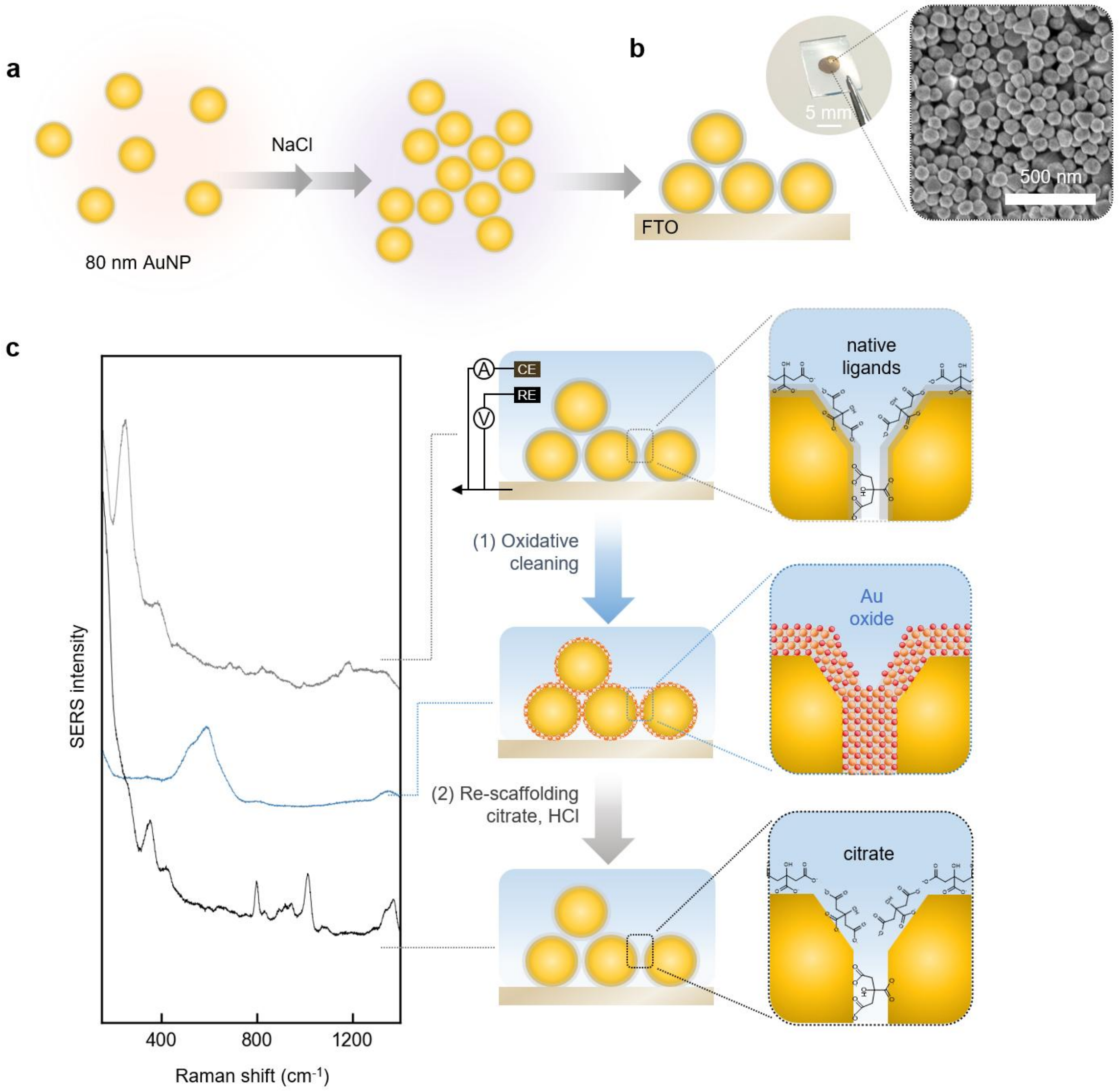

Fig. S2. Preparation, oxidative cleaning, and re-scaffolding of MLagg-citrate substrates. (**a**) Schematic of citrate-stabilised AuNPs aggregated with NaCl and deposited onto a FTO-coated glass slide. (**b**) Photograph of a deposited MLagg film on glass and SEM image showing aggregated AuNPs. (**c**) Schematic illustration of nanogap regeneration: as-prepared MLagg with citrate and other residual surfactants, after electrochemical oxidation in buffer (+1.5 V for 60 s) with formation of a thin Au oxide layer, and after electrochemical re-scaffolding with citrate in HCl (linear sweep reduction from +0.7 V to 0.0 V). Corresponding SERS spectra are shown for each stage.

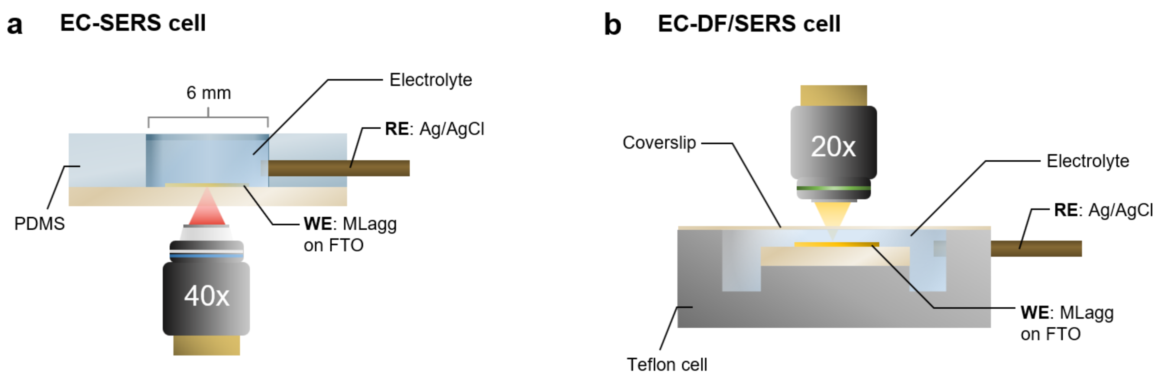

Fig. S3. Spectro-electrochemical cells for static measurements. (**a**) Inverted cell design, where the objective is positioned below the sample and the MLagg is probed from the bottom through the FTO-coated glass substrate. (**b**) Upright cell design, where the objective is positioned above the sample and spectra are measured through a coverslip and electrolyte layer, probing the MLagg from the top.

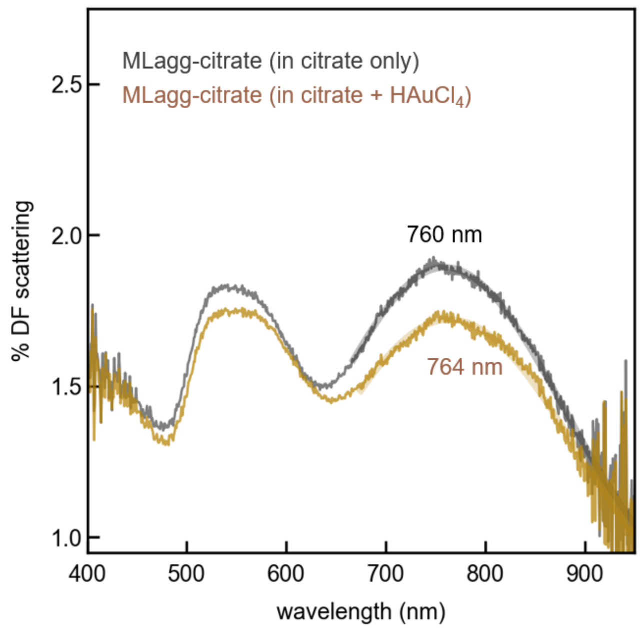

Fig. S4. Dark field scattering spectra of MLagg-citrate before and after adding $HAuCl_4$. MLagg-citrate is initially incubated in 1 mM citrate/HCl at pH 5 (black line). $HAuCl_4$ is injected to a final concentration of 0.1 mM (gold line), resulting in a decrease in scattering intensity and a red shift in the coupled plasmon mode from 760 nm to 764 nm.

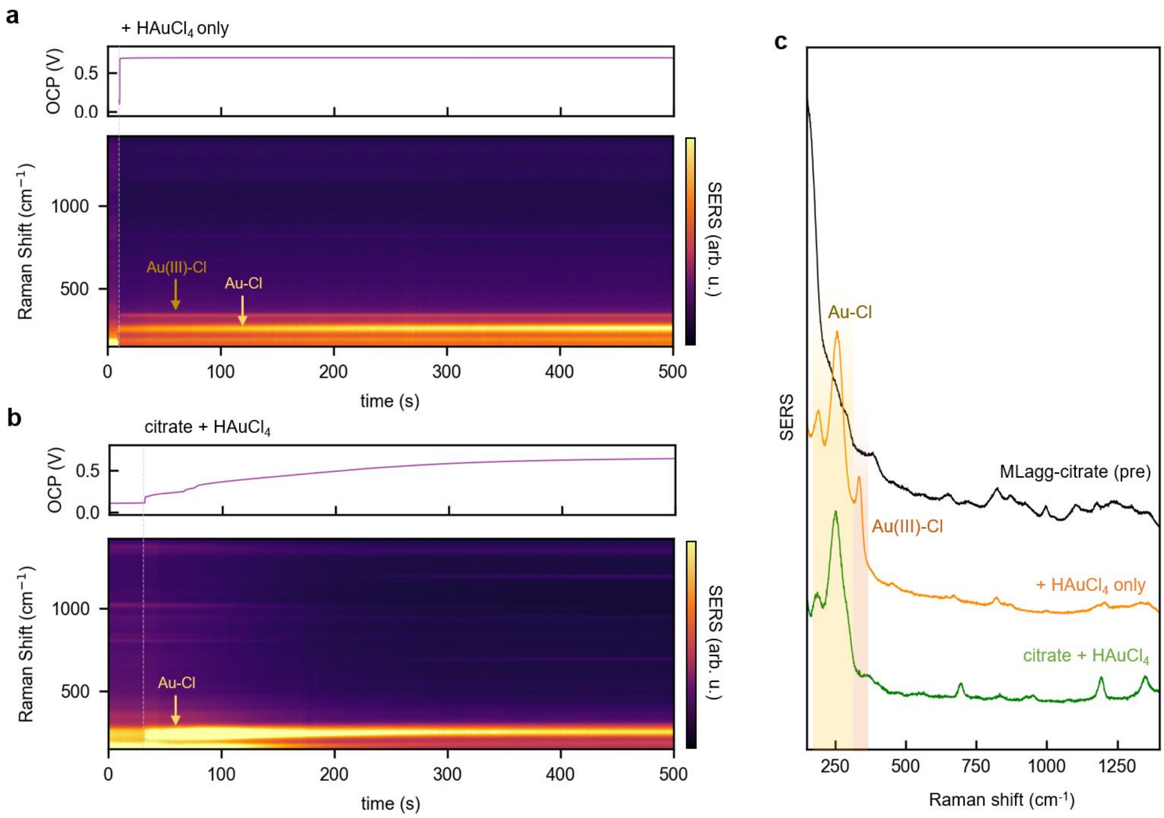

Fig. S5. MLagg-citrate in $HAuCl_4$ with and without citrate. Time-series SERS and OCP of MLagg-citrate incubated in (**a**) 0.1 mM $HAuCl_4$ and HCl (pH 5-6) only, or (**b**) initially in 1 mM citrate and HCl (pH 5-6), followed by injection of 0.1 mM $HAuCl_4$. (**c**) Offset SERS spectra comparing MLagg-citrate before and after treatment with HAuCl4 alone versus citrate+$HAuCl_4$. Without excess citrate in solution, the SERS spectrum shows a strong ~350 cm^{-1} band assigned to Au(III)-Cl stretch and a 260 cm^{-1} band from Au-Cl. When citrate is present, the Au(III)-Cl feature is suppressed, indicating that the surface is instead dominated by Au-Cl species.

SI Note 1: Evolution of citrate binding modes during AuNP growth

Citrate binding provides a sensitive probe of surface structure throughout the AuNP growth cycle. **Fig. S6** and **Table S2** highlight the evolution of key citrate vibrations (see **Table S1** for summary of citrate modes), revealing distinct coordination regimes associated with formation, regeneration, and collapse of the Au-Cl adlayer.

Immediately after $HAuCl_4$ addition and formation of the Au-Cl adlayer, citrate features sharpen markedly. This includes the asymmetric COO^- modes (1560, 1590, 1640 cm^{-1}) and C-C vibrations (995, 1020, 1080 cm^{-1}). A 20 cm^{-1} downshift of the bidentate COO^- mode (1380→1360 cm^{-1}) suggests that the Au-Cl surface dipole, which renders neighbouring Au atoms more electron-deficient, subtly reorganises citrate coordination toward a more uniform, stabilised geometry. Notably, the persistent 995 cm^{-1} mode, arising from gap-spanning citrate in the MLagg architecture, serves as an internal reporter of nanogap stability.

During mid-growth, as Au-Cl is regenerated from continued reduction of $HAuCl_4$, citrate modes decrease in intensity, particularly the 1080 cm^{-1} adatom band and bidentate COO^- modes. This behaviour indicates displacement of coordinated citrate by re-forming Au-Cl, consistent with the dynamic Au(III)-Cl→Au(I)-Cl→Au(0) cycle described in the main text. Meanwhile, the rising SERS background reflects continued Au(0) deposition and surface restructuring.

In late growth, the citrate spectrum exhibits pronounced broadening and fluctuations, especially in the 1080 and 1300 cm^{-1} regions associated with adatom-bound and monodentate citrate. These fluctuations point to real-time structural dynamics, including adatom migration, ligand reorganisation, and local plasmonic reshaping, driven by increasing surface disorder as the Au-Cl adlayer destabilised. The concurrent rise in picocavity-type features further supports this, indicating formation of transient atomic-scale structures in the nanogaps.

Immediately after the OCP collapse, citrate bands become highly broadened, consistent with rapid restructuring as Au-Cl is reduced and citrate rebinds to the newly metallic surface. As the surface stabilises, citrate modes gradually sharpen again, signalling re-establishment of a stable citrate-passivated metallic state. These ligand signatures provide independent validation of the interfacial transformations described in the main manuscript and clarify how citrate responds to formation, evolution, and collapse of the Au-Cl adlayer.

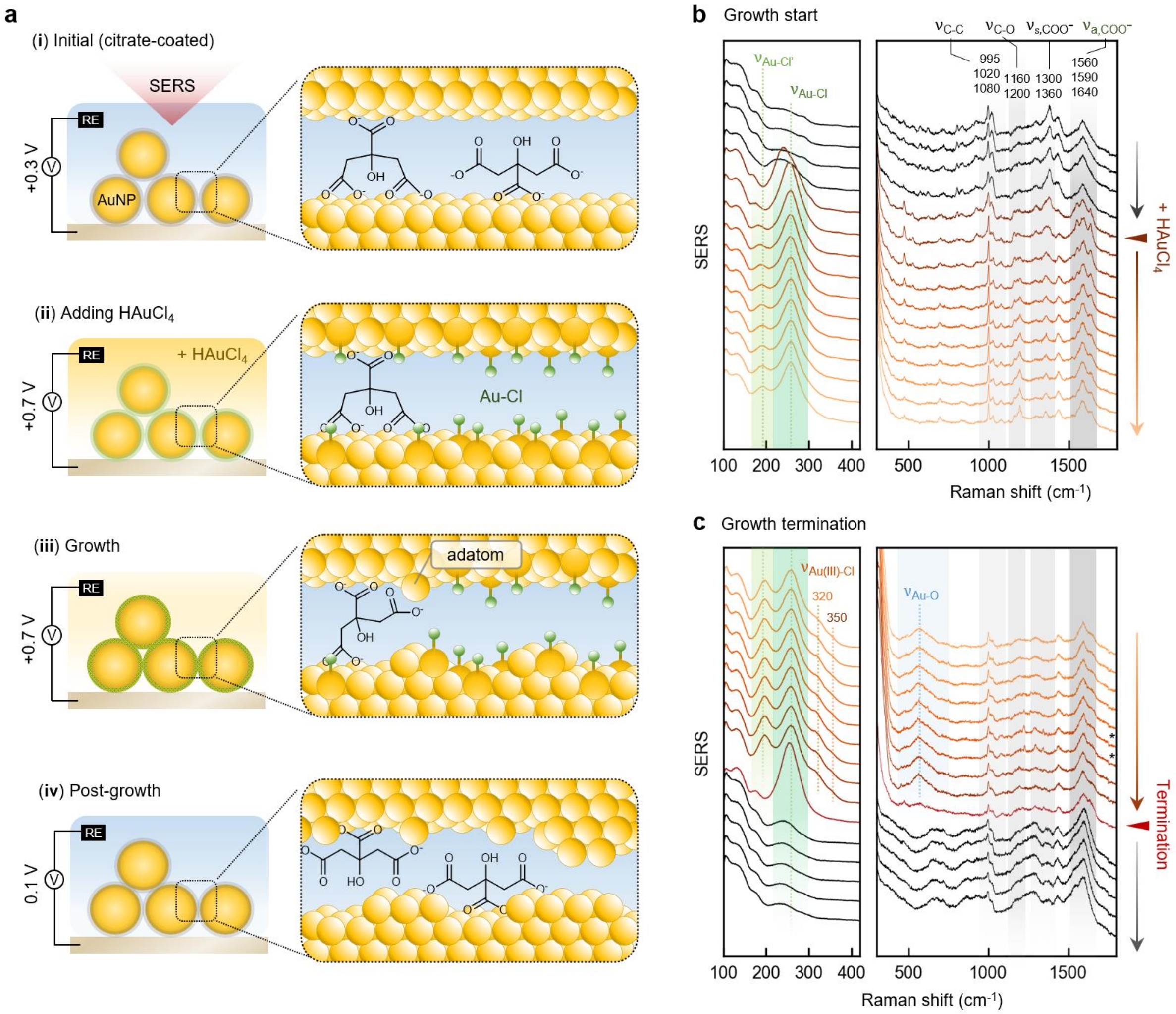

Fig. S6. Interfacial transformations during simulated AuNP regrowth on MLagg-citrate. (a) Shown are four representative stages: (**i**) initial state with citrate molecules coating the nanogap surface; (**ii**) early stage after $HAuCl_4$ injection, where a surface Au-Cl adlayer forms; (**iii**) growth stage, in which Au adatoms deposit at the surface; and (**iv**) post-growth stage, where the transient Au-Cl adlayer is displaced and the surface is again stabilized by citrate. (**b**) SERS spectra at the onset of growth and (**c**) near growth termination highlighting Au-Cl and citrate modes. Key spectral features are labelled. Asterisks (*) highlight spectra exhibiting picocavity-like features.

Table S1 | Raman modes for surface-bound Au species and citrate

Species	Vibration	Mode (cm^{-1})	Remarks	Ref
Au-Cl	$v_{Au-Cl'}$	170-200	DFT of Au-Cl with Cl bonded to Au adatom on Au(111)	8
	v_{Au-Cl}	240-270	DFT of Au-Cl, where Cl is bonded to Au atom in Au(111) slab	8
$AuCl_{(s)}$		96, 168,<br>289 (sharp),<br>341, 367	Raman of solid	8–10
$Au(I)Cl_2^-$	v_{Au-Cl}	327	Raman of solid	11,12
$Au(III)Cl_4^-$	$\delta_{Cl-Au-Cl}$	173	Raman of solid $AuCl_3$<br>SERS of Au(III)-Cl species on Au	8,11,13
	$v_{Au(III)-Cl}$	325, 347		
Au-O	v_{Au-O}	590	Broad; consists of three Au oxide modes at 520, 590, 660 cm^{-1}	8,14,15
citrate	$v_{(C-C)}$	995-1000	Associated with nanogap-spanning modes<br>C_2-t_2t_2, c_2t_2-t_2, where all carboxylate groups are bound to Au	16
		1020	Central carboxylate bound to Au	
		1080	Active when terminal and central carboxylates are bound<br>Enhanced when terminal carboxylate is coordinated to adatom	
	$v_{(C-O)}$	~1200	Possibly C-OH binding to Au	
	$v_{s,(COO^-)}$	1300	Monodentate binding of COO^- to Au	
		1360-1380	Bidentate binding of COO^- to Au	
	$v_{as,(COO^-)}$	1560	Terminal bidentate COO- binding to Au	
		1590	Terminal-central bidentate COO- binding to Au	
		1640	Central bidentate COO- binding to Au	
		Higher modes (weak, 1680)	Associated with monodentate binding with carboxylate unbound	

Table S2 | Summary of citrate-based AuNP regrowth stages. Key spectro-electrochemical observations were tracked per growth stage, with the SERS peak position and intensity changes noted. (Arrows indicate changes in peak intensity: (↑) intensity increase, (↓) intensity decrease, (–) no change in intensity, (↑↑) sharp increase, (↓↓) decrease to become undetectable). Other spectral features such as background (BG) intensity changes are also noted.

Stage		Observations								Notes
		SERS							OCP	
		Au-Cl	Au(III)-Cl, Au-O	citrate				Misc.		
				$\nu_{(C-C)}$	$\nu_{(C-O)}$	$\nu_{s,(COO^-)}$	$\nu_{as,(COO^-)}$			
Seed stage, in citrate		230, weak		995<br>1020	1200<br>(broad, weak)	1300<br>1380	1560 (weak)<br>1590		0.3 V	Citrate bound to nanogaps in multiple modes: gap-spanning and through central carboxylate (c_2), COO- with mono and bidentate binding
Growth start (+ $HAuCl_4$)		↑↑<br>230→260<br>(sharp rise)		– 995<br>↓1020	↑ 1200	– 1300 (sharper)<br>– 1380<br>(broader)	↑ 1560<br>(sharper)<br>↑ 1590<br>(sharper)<br>↑ 1640<br>(sharper)	↓BG	0.3-<br>0.7 V	$AuCl_4^-$ + citrate → Au-Cl<br>Citrate with c_2 binding decreases
Growth phase	I	↓ 260		– 995 (sharper)<br>– 1020 (sharper)<br>↑ 1080	↑ 1200	↓ 1300<br>– 1380 → 1360<br>(sharper)	↑ 1560<br>↑ 1590<br>↑ 1640	↑BG	0.7V	Au-Cl + e- → Au(0)<br>Formation of adatoms from Au reduction<br>Citrate binding onto adatoms
	II	↑ 260		– 995<br>– 1020<br>(broader)<br>↓ 1080	↓ 1200	– 1300<br>↓↓1360	↓ 1550<br>– 1590<br>– 1640	↑BG	0.7V	$AuCl_4^-$ + citrate → Au-Cl<br>Au-Cl + e- → Au(0)<br>Formation of adatoms from Au reduction<br>Loss of 1360 cm^{-1} peak suggests mostly monodentate COO- binding
	III	↓ 260	↑ Au-O<br>↑ Au(III)-Cl<br>[350]	– 995<br>↓1020 (broader)<br>1080 fluctuating	1200<br>fluctuating	– 1300<br>fluctuating	– 1560<br>– 1590<br>↓ 1640	↑BG<br>↑pico-cavities	0.7-<br>0.8V	Au-Cl → Au(0) + Au(III)-Cl<br>(disproportionation)<br>Formation of adatoms from Au reduction, dynamic citrate binding<br>Build-up of Au(III)-Cl and Au-O species
Growth termination		↑↑260<br>(sharp rise)<br>↓260→240	↓↓ Au-O<br>↓↓ Au(III)-Cl	– 995<br>↓ 1020<br>1080 fluctuating	1200<br>fluctuating	– 1300	– 1560<br>↓ 1590<br>– 1640	↑↑BG	0.8-<br>0.1 V	Au(III)-Cl →Au-Cl<br>Au-Cl + citrate → Au(0)<br>Au-Cl layer collapse
Post-growth	I	↓240		– 995<br>– 1020	Part of broad set of peaks 1200-1300	1300 (broader)<br>↑1360	↑1560<br>↑1590<br>↓↓1640	↑BG<br>↑pico-cavities	0.1-<br>0V	Desorption of Cl^-,<br>Broadening of citrate peaks
	II	– 230		– 995<br>– 1020	↑ 1200	– 1300<br>– 1360	↑1560<br>1590 dominant	↓BG<br>↓pico-cavities	0 V	Citrate rearrangement, surface rearrangement

SI Note 2: Limitations of MLagg-citrate model

The MLagg-citrate system provides direct spectroscopic access to interfacial redox and ligand-exchange processes that are challenging to monitor during colloidal AuNP synthesis. However, several system-specific limitations should be considered when relating these observations to classical Turkevich growth.

(1) Nanogap confinement.
The MLagg architecture contains sub-nanometre gaps between adjacent AuNPs, creating confined environments with restricted diffusion and enhanced electric fields. These nanogaps can influence ligand behaviour: for example, the persistent 995 cm^{-1} citrate band reflects gap-spanning coordination that is not expected in dispersed colloids. In colloidal systems, where particle surfaces are fully accessible, citrate adsorption is likely more uniform across facets, leading to different binding equilibria and less persistent gap-specific modes.

(2) Room-temperature, static conditions.
Typical Turkevich syntheses occur at 90-100°C with continuous convection, promoting rapid precursor transport, ligand mobility, and surface restructuring. The MLagg model, operated at room temperature without flow, likely slows key processes such as Au-Cl replenishment and reduction. As a result, some intermediate states may persist longer than in colloidal reactions, and kinetic barriers to structural rearrangement may appear more pronounced.

(3) Optical perturbation.
Spectro-electrochemical measurements necessarily involve laser excitation, which can introduce local photothermal heating or light-driven effects. These were mitigated by minimizing exposure during kinetic scans, but residual perturbation cannot be fully excluded. Control experiments without illumination remain important for distinguishing thermal and electrochemical contributions.

Despite these differences, the MLagg-citrate platform reproduces the sequence of interfacial transformations, such as the formation, evolution, destabilisation, and collapse of the Au-Cl adlayer, observed indirectly in classical AuNP growth. Its strength lies in revealing these transitions with molecular specificity and time resolution that are inaccessible in colloidal suspensions. Future adaptations incorporating temperature control, flow, or colloid-surface hybrids may help bridge the gap between immobilised and freely dispersed nanoparticle environments.

SI Note 3: Preliminary AuNP synthesis with $AuBr_4^-$

The standard synthesis of AuNPs by the Turkevich method uses a $AuCl_4^-$ salt (H, or Na) as the gold source. It was thought that an alternative precursor could provide better insights into the role of Cl^- and its species on the growth of AuNPs. To this end, a comparison was made between AuNPs prepared using $HAuCl_4$ and $HAuBr_4$ as the gold precursors. While a recent work did compare these metal precursors[17], this involved a non-Turkevich synthesis of AuNPs. The redox reactions and redox potentials[18,19] are shown below:

$$AuCl_4^- + 3e^- \rightleftharpoons Au + 4Cl^- \qquad E^0 = 1.002\ V$$

$$AuBr_4^- + 3e^- \rightleftharpoons Au + 4Br^- \qquad E^0 = 0.854\ V$$

As the redox potential for $AuCl_4^-$ is larger, it is more easily reduced. However, for the sake of comparing the precursors, the same reaction conditions were used for both. In an initial attempt, a citrate solution was added to a boiling solution of the gold precursor. This was found to not work for $HAuBr_4$ precursor as under the conditions tested, no stable AuNPs were formed. When the process was reversed, and the gold precursor solutions added to a boiling citrate solution, more stable AuNPs were formed. However, by visual inspection (**Fig. S7a**) and extinction spectroscopy (**Fig. S7b**), the resulting AuNPs had very different optical properties. Unlike the AuNPs prepared using $HAuCl_4$, the AuNPs made with $HAuBr_4$ had a distinct brown colour, and broad optical features across the visible and near-infrared. These broad optical features were attributed to the wide diversity of nanostructures that were observed by scanning electron microscopy (**Fig. S8**). Although determining the precise role of Br^- in the synthesis is outside the scope of this initial work, drawing parallels to gold nanorods where cetyltrimethylammonium bromide reacts with $AuCl_4^-$ to form $AuBr4^-$, it appears that Br^- influences the directionality of the growth even when citrate is used as the reducing and stabilizing ligand. As opposed to forming more isotropic nanostructures as $HAuCl_4$ does, substituting with $HAuBr_4$ yields anisotropic nanostructures, including nanorods and nanoplates. Au nanoplates were also observed in the previously mentioned non-Turkevich synthesis[17].

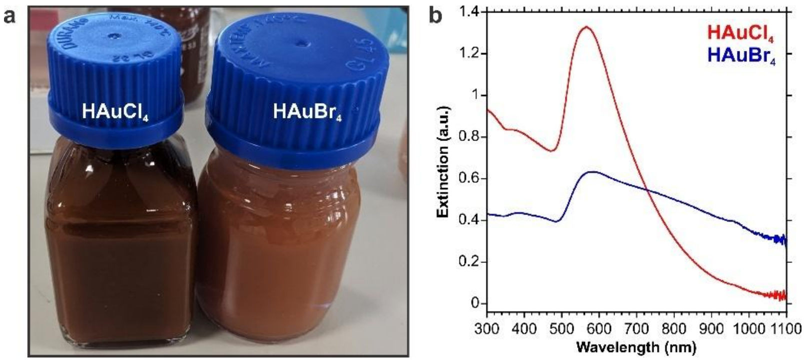

Fig. S7. Comparison of AuNPs prepared by $HAuCl_4$ and $HAuBr_4$. (**a**) Optical images of the prepared colloidal solutions. (**b**) Extinction spectra.

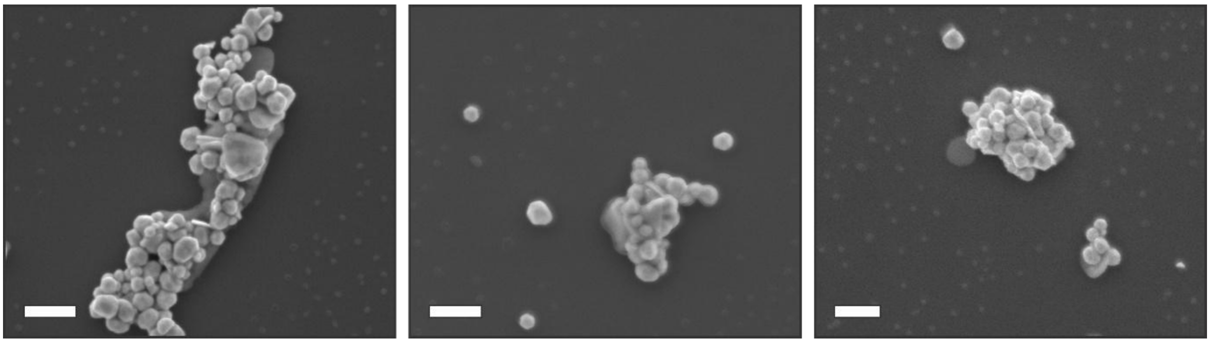

Fig. S8. Scanning electron microscope images of AuNPs prepared using $HAuBr_4$.

A third attempt at the synthesis of the AuNPs was also performed, this time with a higher ratio of citrate to Au, with an intention of forming smaller AuNP seeds. Under these conditions, the AuNPs prepared with $HAuBr_4$ were paler (**Fig. S9a**), and had a weaker extinction (**Fig. S9b**). Interestingly, when the samples were left undisturbed for several days, the optical properties of the $HAuBr_4$ prepared AuNPs noticeably changed while those of the $HAuCl_4$ AuNPs remained similar. Over the course of four days, the previously pale HAuBr4 AuNPs changed to purple (**Fig. S9c**). Although this could have been the result of aggregation, the increase in extinction intensity, red shift of the LSPR with time, and the appearance of a new broad LSPR in the near-infrared (**Fig. S9d,e**), suggest that unlike the $HAuCl_4$ AuNPs, new structures and likely anisotropic growth is occurring. A delayed reduction is somewhat unsurprising given the lower redox potential of $AuBr_4^-$, but the reaction occurring at room temperature is interesting. These phenomena have not been fully explored, and may warrant further investigation in the future.

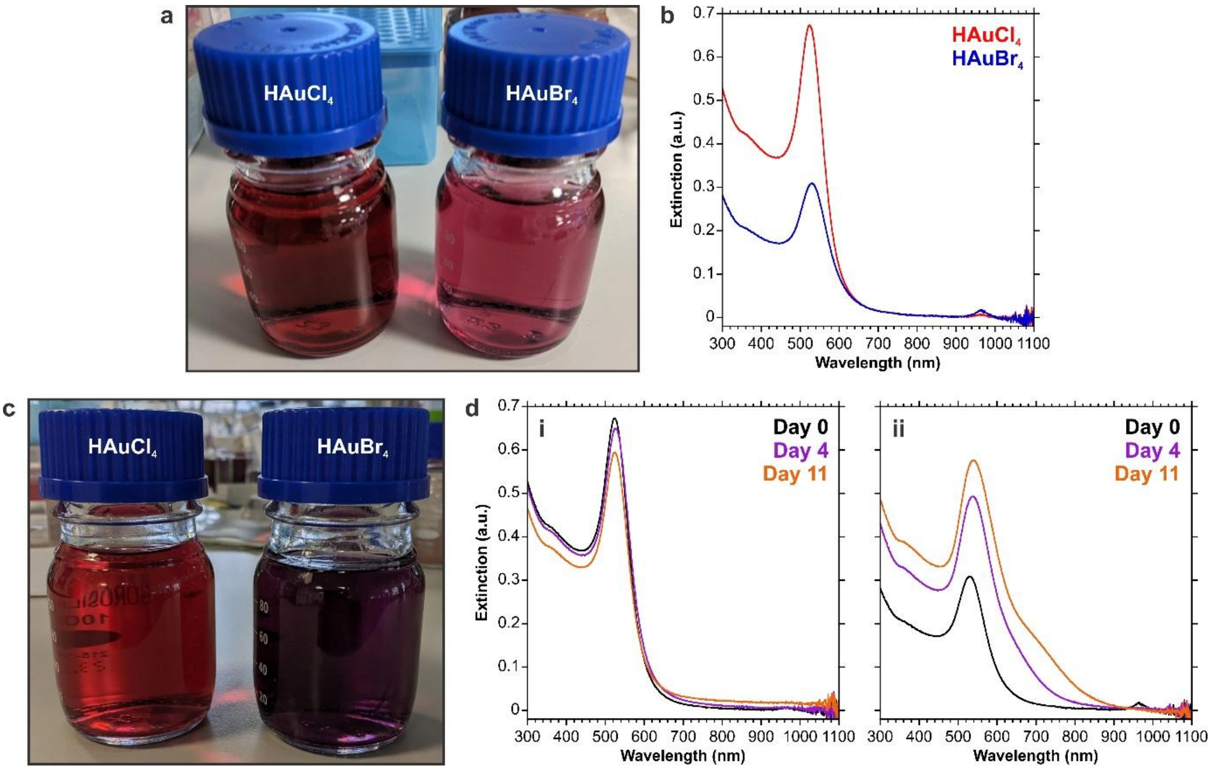

Fig. S9. Comparison of AuNP seeds prepared with $HAuCl_4$ and $HAuBr_4$. (**a**) Optical images of the as-prepared colloidal solutions. (**b**) Extinction spectra of the as-prepared colloidal solutions. (**c**) Optical images of colloidal solutions after aging for four days. (**d**) Extinction spectra of aged colloidal solutions prepared with (**i**) $HAuCl_4$, and (**ii**) $HAuBr_4$.

References

1 D.-B. Grys, M. Niihori, R. Arul, S. M. Sibug-Torres, E. W. Wyatt, B. de Nijs and J. J. Baumberg, *ACS Sens.*, 2023, **8**, 2879–2888.

2 R. Arul, D.-B. Grys, R. Chikkaraddy, N. S. Mueller, A. Xomalis, E. Miele, T. G. Euser and J. J. Baumberg, *Light Sci. Appl.*, 2022, **11**, 281.

3 S. M. Sibug-Torres, D.-B. Grys, G. Kang, M. Niihori, E. Wyatt, N. Spiesshofer, A. Ruane, B. de Nijs and J. J. Baumberg, *Nat. Commun.*, 2024, **15**, 2022.

4 D.-B. Grys, M. Niihori, R. Arul, S. M. Sibug-Torres, E. W. Wyatt, B. De Nijs and J. J. Baumberg, *ACS Sens.*, 2023, **8**, 2879–2888.

5 R. W. Taylor, R. Esteban, S. Mahajan, J. Aizpurua and J. J. Baumberg, *J. Phys. Chem. C*, 2016, **120**, 10512–10522.

6 N. G. Bastús, J. Comenge and V. Puntes, *Langmuir*, 2011, **27**, 11098–11105.

7 J. Polte, *CrystEngComm*, 2015, **17**, 6809–6830.

8 S. M. Sibug-Torres, M. Niihori, E. Wyatt, R. Arul, N. Spiesshofer, T. Jones, D. Graham, B. De Nijs, O. A. Scherman, R. R. Rao, M. P. Ryan, A. Squires, C. N. Savory, D. O. Scanlon, A. Daaoub, S. Sangtarash, H. Sadeghi and J. J. Baumberg, *Nat. Chem.*, 2026, **18**, 294–301.

9 B. Forneris, J. Hiraishi, F. A. Miller and M. Uehara, *Spectrochim Acta Part Mol Spectrosc*, 1970, **26**, 581-591,.

10 E. Husson, N. Quy Dao and D. K. Breitinger, *Spectrochim. Acta Part Mol. Spectrosc.*, 1981, **37**, 1087–1092.

11 K. Watling, G. A. Hope and R. Woods, *J. Electrochem. Soc.*, 2005, **152**, D103.

12 P. Braunstein and R. J. H. Clark, *J Chem Soc Dalt Trans*, DOI:10.1039/DT9730001845.

13 B. H. Loo, *J. Phys. Chem.*, 1982, **86**, 433–437.

14 O. Diaz-Morales, F. Calle-Vallejo, C. De Munck and M. T. M. Koper, *Chem. Sci.*, 2013, **4**, 2334.

15 U. Zhumaev, A. V. Rudnev, J. F. Li, A. Kuzume, T. H. Vu and T. Wandlowski, *Electrochim Acta*, 2013, **112**, 853-863,.

16 D.-B. Grys, B. De Nijs, A. R. Salmon, J. Huang, W. Wang, W.-H. Chen, O. A. Scherman and J. J. Baumberg, *ACS Nano*, 2020, **14**, 8689–8696.

17 M. P. Sørensen, H. Fokam, A. Smolska, J. C. H. Larsen, N. Lock and J. Quinson, *Chemistry*, 2025, preprint, DOI: 10.26434/chemrxiv-2025-l8w54.

18 R. Long, S. Zhou, B. J. Wiley and Y. Xiong, *Chem. Soc. Rev.*, 2014, **43**, 6288.

19 F. Alguacil, *Gold Bull.*, 2006, **39**, 138–138.
